# Metagenomic Shotgun Sequencing Analysis of Canalicular Concretions in Lacrimal Canaliculitis Cases

**DOI:** 10.3390/cimb43020049

**Published:** 2021-07-12

**Authors:** Yukinobu Okajima, Takashi Suzuki, Chika Miyazaki, Satoshi Goto, Sho Ishikawa, Yuka Suzuki, Kotaro Aoki, Yoshikazu Ishii, Kazuhiro Tateda, Yuichi Hori

**Affiliations:** 1 Department of Ophthalmology, Toho University, 6-11-1 Omori-nishi, Ota-ku, Tokyo 143-8541, Japan; t.suzuki@med.toho-u.ac.jp (T.S.); yuka.suzuki@med.toho-u.ac.jp (Y.S.); yhori@med.toho-u.ac.jp (Y.H.); 2 Hyogo Prefectural Amagasaki General Medical Center, 2-17-77 Higashi-nanba cho, Amagasaki 661-0892, Japan; chika@tcct.zaq.ne.jp; 3 Department of Ophthalmology, School of Medicine, The Jikei University, 3-19-18 Shinbashi-nishi, Minato-ku, Tokyo 105-8471, Japan; satosatoshi23@gmail.com; 4 Department of Ophthalmology, School of Medicine, Saitama University, 38 Morohongo Moroyama-machi, Iruma-gun, Saitama 350-0495, Japan; sho_ijp@yahoo.co.jp; 5 Department of Microbiology and Infectious Diseases, School of Medicine, Toho University, 6-11-1 Omori-nishi, Ota-ku, Tokyo 143-8541, Japan; kotaro.aoki@med.toho-u.ac.jp (K.A.); y.ishii58@gmail.com (Y.I.); kazu@med.toho-u.ac.jp (K.T.)

**Keywords:** metagenome, lacrimal canaliculitis, canalicular concretions, polymicrobial infection

## Abstract

Lacrimal canaliculitis is a rare infection of the lacrimal canaliculi with canalicular concretions formed by aggregation of organisms. Metagenomic shotgun sequencing analysis using next-generation sequencing has been used to detect pathogens directly from clinical samples. Using this technology, we report cases of successful pathogen detection of canalicular concretions in lacrimal canaliculitis cases. We investigated patients with primary lacrimal canaliculitis examined in the eye clinics of four hospitals from February 2015 to July 2017. Eighteen canalicular concretion specimens collected from 18 eyes of 17 patients were analyzed by shotgun metagenomics sequencing using the MiSeq platform (Illumina). Taxonomic classification was performed using the GenBank NT database. The canalicular concretion diversity was characterized using the Shannon diversity index. This study included 18 eyes (17 patients, 77.1 ± 6.1 years): 82.4% were women with lacrimal canaliculitis; canalicular concretions were obtained from 12 eyes using lacrimal endoscopy and six eyes using canaliculotomy with curettage. Sequencing analysis detected bacteria in all samples (Shannon diversity index, 0.05–1.47). The following genera of anaerobic bacteria (>1% abundance) were identified: *Actinomyces* spp. in 15 eyes, *Propionibacterium* spp., *Parvimonas* spp. in 11 eyes, *Prevotella* spp. in 9 eyes, *Fusobacterium* spp. in 6 eyes, *Selenomonas* spp. in 5 eyes, *Aggregatibacter* spp. in 3 eyes, facultative and aerobic bacteria such as *Streptococcus* spp. in 13 eyes, *Campylobacter* spp. in 6 eyes, and *Haemophilus* spp. in 3 eyes. The most common combinations were *Actinomyces* spp. and *Streptococcus* spp. and *Parvinomonas* spp. and *Streptococcus* spp., found in 10 cases. Pathogens were identified successfully using metagenomic shotgun sequencing analysis in patients with canalicular concretions. Canalicular concretions are polymicrobial with anaerobic and facultative, aerobic bacteria.

## 1. Introduction

Lacrimal canaliculitis, an infectious inflammation of the lacrimal canaliculus, often remains undiagnosed for extended periods as a result of its rarity and variety. Patients with lacrimal canaliculitis typically present with red eye, epiphora, and punctal discharge. Lacrimal canaliculitis can be primary or secondary. In 1854, Von Graefe first reported primary canaliculitis, which leads to concretions with lacrimal canaliculus, and in 1878, Israel, who described human Actinomycosis, considered it to be often associated with *Actinomyces* species (spp.). Secondary canaliculitis is often a complication of punctal or intercanalicular plug insertion for treating dry eye [1].

The presence of concretions may shield the bacteria from the antibiotics [2], thus promoting resistance and an inadequate treatment response. Hence, early diagnosis and prompt surgical management are important. Many studies have summarized the clinical characteristics and treatment of primary canaliculitis, but few have investigated primary canaliculitis with concretions.

Previous reports on the culture of lacrimal duct stones showed that *Actinomyces* was detected in 5% to 44.4% [3,4,5,6,7,8,9,10], while pathologic evaluation showed a discrepancy of more than 90%, depending on the test. The reasons for this are that traditional bacterial culture and identification are both time-consuming and laborious, while some anaerobic bacteria can be difficult to isolate and identify.

Consequently, the history of canalicular concretion studies is long, and the pathophysiology remains unknown. However, metagenomic shotgun sequencing analysis using next-generation sequencing (NGS) was introduced recently and enables the identification of all bacterial species with their taxonomic classification [11,12,13,14,15,16]. This method has been applied successfully to detailed analysis of the microbiome. The term NGS is generically used to indicate the two main sequencing methods: the marker gene sequencing approach (targeted-amplicon sequencing) and the shotgun approach. Targeted-amplicon sequencing is used mainly in microbiome analysis with taxonomic purposes. However, the shotgun approach to sequencing is performed across random fragments of all DNA in a given sample and also can be used in cases with an unknown microbial target [15]. In addition, shotgun metagenomics facilitates the simultaneous study of viruses, bacteriophages, archaea, and eukaryotes [17]. NGS does not require target-specific primers; the entire genome of a pathogen is sequenced randomly and directly from clinical samples. As a result, some studies have used NGS for pathogen identification in culture-negative cases [18]. Li et al. [11] reported 16 cases of infectious keratitis and four controls in formalin-fixed corneas analyzed by NGS to identify pathogens from corneal specimens. Doan et al. [19] reported that metagenomic DNA sequencing was highly concordant in cases of uveitis and was superior to pathogen-directed polymerase chain reaction. These previous studies established the feasibility of using metagenomics to investigate bacteria, fungi, amoeba, and viruses associated with pathogenic ocular infections.

Therefore, we believe that NGS is the best method for biome analysis of canalicular concretions. No studies have reported the use of NGS to detect the pathogens causing canalicular concretions with lacrimal canaliculitis. In this first study, we describe the results of using NGS for metagenomic shotgun sequencing analysis of canalicular concretion samples from primary lacrimal canaliculitis; this is the first report to clarify the composition of the canalicular concretions.

## 2. Materials and Methods

### 2.1. Subject Recruitment

This prospective study was approved by the Ethics Committee of the Faculty of Medicine, Toho University School of Medicine (No. 27019) and was conducted in accordance with the principles of the Declaration of Helsinki. Written informed consent was obtained from all the studied subjects for sample collection and subsequent analyses.

### 2.2. Sampling of Canalicular Concretions

We investigated patients with primary lacrimal canaliculitis who were examined in Toho University Omori Medical Center Hospital, Amagasaki Medical Center Hospital, Jikei University Hospital, Saitama Medical Center Hospital from February 2015 to July 2017. Exclusion criteria for this study were secondary canaliculitis such as punctal plug placement, facial surgery or trauma, allergies, and recurrent cases. In this study, we analyzed the pathogens of canalicular concretions. Because lacrimal stones (concretion or dacryoliths) can occur in any part of the lacrimal system, we enrolled only patients with primary canalicular concretions in this study.

Specimens such as canalicular concretion were collected and they were placed in Eppendorf tubes and stored in a freezer at −80 °C until DNA extraction. All specimens were frozen and sent to the Department of Microbiology and Infectious Diseases at the Faculty of Medicine, Toho University School of Medicine.

### 2.3. DNA Extraction and Shotgun Metagenomic Sequencing Analysis

After the specimens were treated with achromopeptidase, nucleotides were extracted using a Recover All Total Nucleic Acid Isolation Kit for FFPE (Life Technologies, Thermo Fisher Scientific Inc., Waltham, MA, USA). Nextra XT DNA Library Preparation Kit (Illumina, Inc., San Diego, CA, USA) was used in preparation for shotgun sequencing DNA libraries. Shotgun metagenomic sequencing for 150 bases of the single end was performed using a MiSeq platform (Illumina, Inc.). Skewer (version 0.1.126) was used for trimming the adapter sequence to less than a Phred quality score (Q) of 15 for low-quality sequences [20]. Human genome sequences were subtracted using the Burrows-Wheeler Aligner with the “MEM” option with the human genome GRCh37.p13 (GenBank assembly accession: GCA_000001405.14) as a mapping reference and SAM tools (version 1.3) [21,22]. Reads with human genome subtracted were analyzed by a MEGABLAST search against the GenBank nt and WGS databases (ftp://ftp.ncbi.nlm.nih.gov/blast/db/) (Access date: 12 May 2016), downloaded in May 2016, followed by metagenomic browser MEGAN5 (http://ab.inf.uni-tuebingen.de/software/megan5) (Access date: 10 September 2015) [23]. The human genome was subtracted from the whole genome, and the subtracted genome was considered the microbial genome (bacterial, eukaryotic, and viral genome). Effective bacterial genera were defined as having an abundance >1.0%.

## 3. Results

### 3.1. Patients’ Characteristics

Seventeen patients (18 eyes; 82.4% women patients) were enrolled in this prospective study. The mean age at presentation was 77.1 ± 6.1 years (range, 68–89 years). In six cases, the canalicular concretion was on the upper right side, in one case on the lower right side, in five cases on the upper left side, and in six cases on the lower left side. The sample collection method using lacrimal passage endoscopy was performed in 12 cases and in six cases canaliculotomy with curettage. Before sample collection, antibacterial eye drops were used in all samples, and in 6 of 16 cases, antibacterial internal drugs were used (Table 1). Microbiology laboratory culture results of removed canalicular concretion are shown (Table 2).

### 3.2. Metagenomic Shotgun Sequencing Analysis

Samples from canalicular concretion from primary lacrimal canaliculitis underwent metagenomic shotgun sequencing analysis. Although most reads were of human origin, nonhuman quality-filtered microbial sequence reads/sample were obtained for subsequent analysis. The proportion of sequence reads with significant hits to bacteria, viruses, fungi, and Eukaryota are summarized in Table 3. In total, the genome minimal number of reads/sample was 27,552; the maximal number was 1,148,458 (average ± standard deviation (SD) 403,011 ± 371,050). Overall, 18 of 18 cases were bacteria-positive (minimal to maximal range detected, 21,000 to 698,597 reads; relative abundance, 18.6–92.1%), virus-positive in 2 of 18 cases (maximal, 33 reads), and fungus-positive 14 of 18 cases (maximal 10,061 reads). All cases were Eukaryota-positive and detected in 91 to 3524 reads.

### 3.3. Taxonomy of Canalicular Concretions and Identification of Bacterial Phyla and Shannon Index

**Figure 1:**
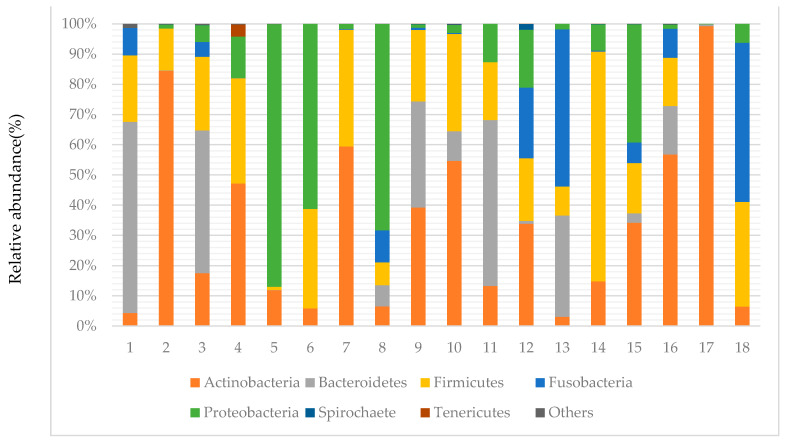
Distribution of bacteria phyla identified by metagenomic analysis of 18 samples obtained from canaliculitis concretions.

Figure 1 shows that the most abundant phyla were *Firmicutes* (26.2%), *Bacteroidetes* (26.1%), *Actinobacteria* (18.1%), *Proteobacteria* (16.8%), and *Fusobacteria* (12.4%), accounting for 99.6% of the total reads. The remaining two phyla, in relatively low abundance (0.4% total), were *Spirochaete* and *Tenericutes*. The most prevalent phyla were *Actinobacteria*, which were found in all 18 samples. *Firmicutes* and *Proteobacteria* were the second most prevalent, detected in 17 samples; *Bacteroidetes* was detected in 16 samples; *Fusobacteria* was detected in 14 samples. The mean Shannon index was 0.97 ± 0.362 (range, 0.047–1.472).

### 3.4. Taxonomy of Canalicular Concretions and Identification of Bacterial Genera

Table 4 provides the number of detected genera in each sample and the number of detected bacterial genera with an abundance >1.0%.

**Figure 2:**
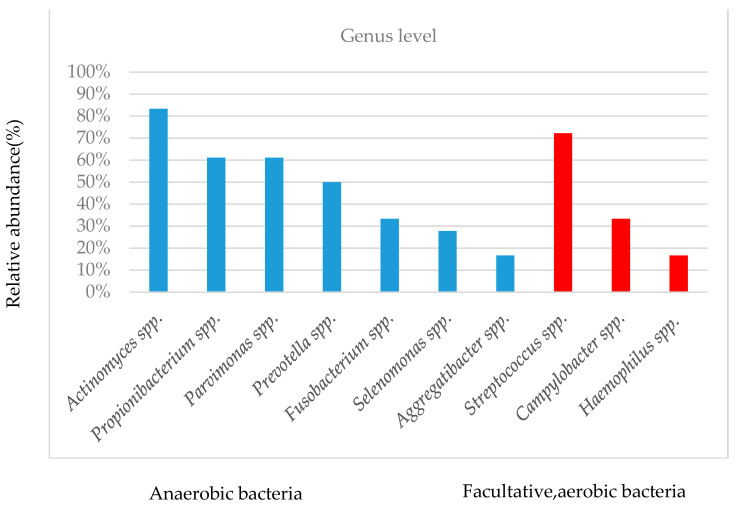
Top 10 bacterial species with the most frequent appearance.

Among the anaerobic bacteria, the most prevalent genera were *Actinomyces* spp. (15/18 eyes, relative abundance, 1.1–54.1%), *Propionibacterium* spp. (11/18 eyes, relative abundance, 5.5–97.9%), *Parvimonas* spp. (11/18 eyes, relative abundance, 2.5–35.4%), *Prevotella* spp. (9/18 eyes; abundance, 2.8–52.7%), *Fusobacterium* spp. (6/18 eyes, abundance, 4.6–52.5%), *Selenomonas* spp. (5/18 eyes, abundance, 1.0–18.6%), and *Aggregatibacter* spp. (3/18 eyes, abundance, 1.1–5.7%). In facultative, aerobic bacteria, the most prevalent genera were *Streptococcus* spp. (13/18 eyes, relative abundance, 2.3–61.9%), *Campylobacter* spp. (6/18 eyes, relative abundance, 3.2–18.5%), and *Haemophilus* spp. (3/18 eyes, relative abundance, 1.0–78.9%). The top 10 species were classified as anaerobic or facultative, aerobic bacteria, as shown in Figure 2. The relative abundance of the prevalent genera varied significantly depending on individual and sample types.

The most common combinations were *Actinomyces* spp. and *Streptococcus* spp. in 10 eyes, *Parvinomonas* spp. and *Streptococcus* in 10 eyes, followed by *Actinomyces* spp. and *Parvinomonas* spp., *Actinomyces* spp. and *Prevotella* spp., *Actinomyces* spp. and *Propionibacterium* spp. (combination of two genera), and *Actinomyces* spp. and *Parvinomonas* spp. and *Streptococcus* spp. (combination of three genera), found in eight eyes.

### 3.5. The Treatment Results

After specimen collection, almost all of the canalicular concretions were removed by lacrimal endoscopy or canaliculotomy with curettage. Recurrence of canaliculitis was observed in one eye in sample 7 (Table 5).

## 4. Discussion

Although lacrimal canaliculitis has been analyzed in culture and pathology, this is the first report using metagenomic shotgun sequencing analysis. The results of our metagenomic shotgun sequencing analysis were used to investigate the canalicular concretions in primary canaliculitis. All evaluated canalicular concretions were bacteria-positive and a few samples were virus- and fungi-positive.

Few studies have investigated the use of metagenomic shotgun sequencing analysis to detect the pathogens causing lacrimal infection such as canaliculitis.

In the current study, *Firmicutes* was the most dominant phylum. In addition, *Bacteroidetes*, *Actinobacteria*, *Proteobacteria*, and *Fusobacteria* were present in abundant levels. *Actinobacteria* was detected in all samples. No previous reports have used NGS to analyze canalicular concretions. The results of conjunctival sac culture [24,25,26] consisted of *Proteobacteria*, *Firmicutes*, and *Actinobacteria* detected at the phylum level. These similar structures were not found in ocular diseases and were similar to the microbiome structure of endodontic diseases in the oral cavity [27]. It is anatomically relevant that the lacrimal drainage system comprises the puncta, canaliculi, lacrimal sac, and nasolacrimal duct, the last of which opens into the inferior nasal meatus [28].

At the gene level, metagenomic analysis showed that the average detected number of bacteria was 5.7, assuming that 1% or more was detected. Comparing the present results with the NGS results, few studies have reported on canalicular concretions, but in dacryocystitis [29], *Staphylococcus*, *Streptococcus*, *Veillonella*, *Haemophilus*, and *Stenotrophomonas* were detected. Comparing the current results with the culture pathological (only *Actinomyces* spp.) results, anaerobic bacteria such as *Actinomyces* spp. were detected in 15 eyes, while in the culture results, *Actinomyces* spp. was detected in 5% to 44.4% [3,4,5,6,7,8,9,10], and in the pathological results was detected in 93.8% to 100% [9,30]; *Propionibacterium* spp. was detected in 11 eyes, but in the culture results, it was undetectable; *Parvimonas* spp. was detected in 11 eyes, but in the culture results, it was undetectable; *Prevotella* spp. in nine eyes and 2.2% in culture results [5]; *Fusobacterium* spp. in six eyes and 28.6% in culture results [10]; *Selenomonas* spp. in five eyes, and *Aggregatibacter* spp. in three eyes, but in culture results, it was undetectable. Facultative bacteria such as *Streptococcus* spp. were detected in 13 eyes, but culture results showed 10.6% to 60% [3,5,8,10]. Aerobic bacteria such as *Campylobacter* spp. were detected in six eyes but were undetected in the culture results. *Haemophilus* spp. was detected in three eyes and was found at 22.2% to 28.6% [3,10] in culture results. In contrast, *Staphylococcus* spp. detected by culture tests ranged from 10% to 53.3% [3,4,5,6,7], while none was detected in our NGS results. The difference between these results is that antimicrobial eye drops and oral antimicrobial medication were used in almost all cases, and anaerobic media with a 5% carbon dioxide atmosphere was used for at least 4 to 6 days, as described by Hussain et al. [10] and Briscoe et al. [9].

The most prevalent genus was *Actinomyces* spp. For a long time, *Actinomyces* spp. was the most common pathogen in lacrimal canaliculitis [10]. Most published studies and case reports on canaliculitis have detailed an attempt to determine the causative organism either by culture or histopathological examination. Perumal et al. [30] reported that they often present in histopathological sections of canalicular concretions and further found that a relatively large percentage of filamentous bacteria (*Actinomyces* spp.) and non-filamentous bacteria (both Gram-positive and Gram-negative) can be seen on pathological analysis. They also proposed that canalicular concretions harbored mixed infections seen on histopathologic analysis. Similarly, *Actinomyces* spp. was a mixed-bacterial infection that coexisted with aerobic and anaerobic bacteria such as in internal medicine diseases [31] and periodontitis [32].

Similarly, *Streptococcus* spp. also has been detected in culture in from 10% to 60% [3,5,7,8,9], which is consistent with our results. *Streptococcus* spp. are clinically important Gram-positive bacteria that can cause a wide variety of diseases in human. The initial step in establishing a bacterial infection is adhesion of the organism to the epithelium of the host. *Streptococcus* spp. use multiple adhesins to attach to host cells [33].

Although *Propionibacterium* spp. is a common ocular surface microflora and may not be recognized as a pathogen because it has been non-pathogenic until now, it has been detected in endophthalmitis [34,35], suggesting either that it may not have been considered a causative agent or that it can become pathogenic by coexisting with other bacteria [36].

According to our metagenomic analysis, common bacterial combinations were *Actinomyces* spp. and *Streptococcus* spp., *Parvinomonas* spp. and *Streptococcus* spp. found in 10 cases, followed by *Actinomyces* spp. and *Parvinomonas* spp., *Actinomyces* spp. and *Prevotella* spp., *Actinomyces* spp. and *Propionibacterium* spp., and the triple combination of *Actinomyces* spp., *Parvinomonas* spp., and *Streptococcus* spp. in eight cases.

Furthermore, when the bacteria detected by metagenomic analysis and bacterial combinations were examined in detail, interestingly, they were similar to those found in oral gingivitis and periodontitis, as well as at the phyla level.

Periodontal diseases occur when a bacterial biofilm (dental plaque) adheres to the boundary between the teeth and gingiva, causing chronic inflammation and progressively destroying the periodontal tissue that supports the teeth. This allows the first bacteria (early colonizers), such as *Streptococcus* spp., to attach to the teeth, colonize, and grow. After some growth of early colonizers, the biofilm becomes more compliant with other bacterial species, known as late colonizers such as *Actinomyces* spp., *Fusobacterium* spp., *Prevotella* spp., *Parvimonas* spp., and *Aggregatibacter* spp. This provides the conditions for cell–cell interactions of both anaerobic bacteria and facultative, aerobic bacteria. In brief, periodontitis is caused by mixed-species communities rather than by individual pathogens working in isolation [37]. Bacterial biofilms cause chronic infections because they show increased tolerance to antibiotics and disinfectant chemicals as well as resisting phagocytosis and other components of the body’s defense system [38]. Above all, *Actinomyces* spp. and *Prevotella* spp. maintain or establish biofilm complexity [39]. It is also important that, anatomically, the lacrimal duct connects the ocular surface to the nose.

The current findings highlight the important role of anaerobic bacteria in canalicular concretions. We hypothesized that lacrimal canaliculitis results from a combination of bacteria (anaerobic and facultative, aerobic) in a closed environment of lacrimal canaliculi, and matures to form a biofilm. The formation of biofilms by bacteria may lead to drug resistance and the development of intractable diseases.

Furthermore, as a result of metagenomic analysis, a small amount of fungi (1.04%) also was detected in addition to bacteria. Recently, a strong association was reported between the presence of *Candida* and periodontal diseases [40]. Vécsei et al. [8] also reported fungi in 23% of cases in culture. We suggested that a few cases of canalicular concretions formed by co-infection with bacteria and fungi.

Lacrimal canaliculitis is often delayed in detection due to a lack of symptoms [2]. Recent studies using optical coherence tomography (OCT) have been able to detect several anatomical elements of the lacrimal gland (ducts, lobules, parenchyma, and acini) [41]. In addition, it has been observed by OCT and other techniques that lacrimal duct obstruction causes changes in tear fluid dynamics [42,43]. This may help in the diagnosis of lacrimal duct inflammation such as canaliculitis by using OCT in the future, and is expected to be a subject of future research.

In the present case, only one of the 18 eyes had recurrence. We believe that the results of shotgun sequencing do not contribute to the choice of antibiotics for the treatment of lacrimal canalicular concretions. The results of this study suggest that canaliculitis is often caused by anaerobic bacteria, and canaliculotomy with curettage or using lacrimal endoscope is a better approach than antibiotic therapy.

Periodontal disease has been associated with systemic vascular diseases such as cerebral infarction and myocardial infarction [44]. If the bacterial composition of lacrimal canaliculitis is similar to that of periodontal disease, it is necessary to elucidate the pathophysiology and establish therapeutic agents.

The results of the current study should be interpreted with caution because of some limitations, one of which is that DNA sequencing alone cannot detect RNA viruses (e.g., rubella). Another limitation was the small sample size. A major limitation of metagenomic analysis is the chance that the results would represent contaminants. In addition, it was difficult to determine if the organisms detected in the metagenomic analysis were pathogenic or nonpathogenic agents. We defined the causative organisms as those detected more than 1% of the time, but there was no cut-off value. Finally, we did not conduct a chemical analysis of the composition of the canalicular concretions, such as calcium, or proteins, such as trefoil factor peptides and mucins.

## 5. Conclusions

Pathogens were identified successfully using metagenomic shotgun sequencing analysis in patients with canalicular concretions. Canalicular concretions are polymicrobial with anaerobic and facultative, aerobic bacteria.

## Figures and Tables

**Table 1:** Patient characteristics.

	Sample
Mean age (years ± SD), *n* = 18	77.1 ± 6.1
No. women (%), *n* = 18	14 (82.4%)
Side of involvement Upper right; lower right; upper left; lower left, eyes (%)	6 eyes; 1 eye; 5 eyes; 6 eyes
Sample collection method using lacrimal passage endoscope or curettage, eyes (%) *n* = 18	12 eyes; 6 eyes
Before sample collection, antibacterial eye drops were used, eyes (%) *n* = 18	18/18
Before sample collection, antibacterial internal drugs were used, eyes (%), *n* = 16 (unknown in 2 eyes)	6/16

**Table 2:** Summary of microbiology laboratory culture of canalicular concretions.

Sample	Microbiology Laboratory Culture
1	*Prevotella melaninogenica*, *Fusobacterium nucleatum*, *Streptococcus milleri*
2	*Actinomyces* spp., *Streptococcus constellatus*
3	No culture test
4	*Actinomyces odontolyticus*, *Eikenella corrodens*
5	Actinomyces spp. *Haemophilus parainfluenzae*
6	*Streptococcus* spp.
7	*Peptostereptococcus* spp., α-*streptococcus*
8	Negative culture for anaerobic bacteria
9	Negative culture for anaerobic bacteria
10	CNS, *Coynebacteirum* spp.
11	No culture test
12	*Propionibacterium* spp.
13	No culture test
14	*Streptococcus milleri* group *Peptostreptococcus* spp.
15	*Haemophilus influenzae*
16	*Haemophilus influenzae*
17	*Actinomyces israelii*
18	*Streptococcus milleri* group, CNS

**Table 3:** Read summary of metagenomic shotgun sequencing analysis of canalicular concretions.

Sample	Total Genome	Bacteria	%	Virus	%	Fungi	%	Eukaryota	%
1	968,005	698,597	72.2	0	0	10,061	1.04	1603	0.17
2	118,762	26,548	22.4	0	0	13	0.01	1938	1.63
3	974,992	181,299	18.6	0	0	120	0.01	1803	0.18
4	166,885	58,750	35.2	0	0	21	0.01	959	0.57
5	77,720	48,107	61.9	0	0	10	0.01	1758	2.26
6	260,161	222,246	85.4	0	0	0	0	2340	0.90
7	103,639	55,698	53.7	0	0	15	0.01	1546	1.49
8	180,213	129,314	71.8	0	0	0	0	91	0.05
9	1,087,307	451,326	41.5	0	0	143	0.01	760	0.07
10	209,085	67,158	32.1	0	0	41	0.02	1496	0.72
11	1,148,458	501,681	43.7	0	0	110	0.01	693	0.06
12	191,096	118,846	62.2	0	0	15	0.01	1553	0.81
13	78,334	65,926	84.2	0	0	0	0	1352	1.73
14	273,766	140,082	51.2	0	0	34	0.01	3524	1.29
15	413,328	153,523	37.1	33	0.01	63	0.02	2317	0.56
16	336,017	126,406	37.6	0	0	35	0.01	1564	0.47
17	27,552	21,000	76.2	2	0.01	2	0.01	2773	10.06
18	638,880	588,396	92.1	0	0	0	0	1243	0.19

**Table 4:** Number of detected genera in each sample and number of detected bacterial genera of 1% or more abundance (ratio of anaerobic bacteria to facultative, aerobic bacteria).

Sample	Number of Detected Bacterial Genera	Number of Detected Bacterial Genera, 1% or More (Ratio of Anaerobic Bacteria to Facultative, Aerobic Bacteria)
1	13	5 (4:1)
2	139	4 (3:1)
3	380	12 (10:2)
4	233	7 (3:4)
5	99	3 (2:1)
6	16	4(2:2)
7	103	5 (4:1)
8	54	7(6:1)
9	109	8 (7:1)
10	204	8 (7:1)
11	82	4 (2:2)
12	68	7 (5:2)
13	45	5 (4:1)
14	95	7 (5:2)
15	157	7 (5:2)
16	119	4 (4:0)
17	21	2 (2:0)
18	15	4(2:2)
Average ± SD	108.4 ± 90.0	5.7 ± 2.3

**Table 5:** The treatment results of canaliculitis.

Sample	Treatment Results
1	No recurrence
2	No recurrence
3	No recurrence
4	No recurrence
5	No recurrence
6	No recurrence
7	Recurrence
8	No recurrence
9	No recurrence
10	No recurrence
11	No recurrence
12	No recurrence
13	No recurrence
14	No recurrence
15	No recurrence
16	No recurrence
17	No recurrence
18	No recurrence

## Abbreviations

CNS	coagulase-negative staphylococci
SD	standard deviation
spp.	species
PCR	polymerase chain reaction
NGS	next-generation sequencing
DNA	deoxyribonucleic acid
RNA	ribonucleic acid
